# Bidirectional coupling of splicing and ATM signaling in response to transcription-blocking DNA damage

**DOI:** 10.1080/15476286.2016.1142039

**Published:** 2016-02-25

**Authors:** Maria Tresini, Jurgen A. Marteijn, Wim Vermeulen

**Affiliations:** Department of Genetics, Cancer Genomics Netherlands, Erasmus University Medical Center, Rotterdam, The Netherlands

**Keywords:** Alternative splicing, ATM, DDR signaling, DNA damage, R-loops, spliceosome, transcription, UV irradiation

## Abstract

In response to DNA damage cells activate intricate protein networks to ensure genomic fidelity and tissue homeostasis. DNA damage response signaling pathways coordinate these networks and determine cellular fates, in part, by modulating RNA metabolism. Here we discuss a replication-independent pathway activated by transcription-blocking DNA lesions, which utilizes the ATM signaling kinase to regulate spliceosome function in a reciprocal manner. We present a model according to which, displacement of co-transcriptional spliceosomes from lesion-arrested RNA polymerases, culminates in R-loop formation and non-canonical ATM activation. ATM signals in a feed-forward fashion to further impede spliceosome organization and regulates UV-induced gene expression and alternative splicing genome-wide. This reciprocal coupling between ATM and the spliceosome highlights the importance of ATM signaling in the cellular response to transcription-blocking lesions and supports a key role of the splicing machinery in this process.

## Introduction

Environmental genotoxins and metabolic byproducts induce a wide variety of DNA lesions which can have detrimental consequences for tissue homeostasis.^1^ Cells have evolved mechanisms to “translate” signals from stochastic DNA damage into organized DNA damage responses (DDR), including activation of repair systems, cell cycle checkpoints and apoptotic programs.^2,3^ DDR is coordinated by signaling networks that utilize posttranslational modifications and protein-protein interactions to elicit the initial stages of the cellular response. Later DDR stages depend largely on modulation of RNA metabolism.

In eukaryotic cells, pre-mRNA splicing toward production of translation-competent mRNAs is a critical stage of RNA metabolism and cumulative evidence supports that it is also an important DDR target.^4^ Damage-induced splicing changes influence the cellular proteome either through production of mis-spliced, rapidly degraded transcripts, or via selective utilization of alternative exons encoding divergent protein domains.^5-7^ How DDR regulates splicing is not yet understood, but in the case of transcription-blocking DNA lesions, splicing changes can be largely attributed to the spatiotemporal coupling between elongating RNA polymerase II (RNAPII) and the splicing machinery. DNA damage alters splicing by modulating the elongation rate of RNAPII,^5^ the interaction between RNAPII and splicing regulators^6,8,9^ or indirectly, through loss of association between late-stage spliceosomes and nascent transcripts.^7^ We have recently reported that this latter mechanism is a two-step process involving a stochastic (cis-) step, triggered by RNAPII pausing at DNA lesions, and a signaling-mediated (trans-) stage, controlled by the Ataxia Telangiectasia Mutated (ATM) DDR kinase.^7^ Intriguingly, the interaction between spliceosome displacement and ATM signaling is reciprocal. Our data support a model by which, displacement of assembled, co-transcriptional spliceosomes from lesion-arrested RNAPII, results in hybridization between free (intron-retaining) pre-mRNA with template ssDNA adjacent to the transcription bubble. The resulting R-loop activates ATM which signals to mobilize spliceosomes in-trans, from elongating polymerases located distal to DNA lesions. In parallel, ATM signals through its canonical pathway to coordinate the cellular DDR.^7^ In this manner, ATM utilizes the RNA splicing machinery to control gene expression and alternative splicing, as to shape the cellular proteome in response to transcription-blocking DNA damage.

This point of view article focuses on this reciprocal regulation between DNA damage-induced spliceosome remodeling and DDR signaling, in the context of transcription-blocking DNA lesions. We will give a brief overview of the current knowledge on reciprocal coupling of transcription with splicing, DNA damage, co-transcriptional R loop formation and ATM signaling, and discuss theoretical considerations of how these processes may influence each other to ensure cellular homeostasis.

### Functional coupling between transcription and RNA splicing

In metazoans, spliceosomes assemble co-transcriptionally and the majority of exons are spliced while the pre-mRNA is still attached to RNAPII.^10^ This spatiotemporal coupling of transcription and splicing is critical during exon selection and operates primarily through two parallel mechanisms: kinetic and recruitment coupling.^10-13^ Recruitment coupling is established by physical associations between splicing regulators and elongating RNAPII, while kinetic coupling is driven by the variable rates of transcription and does not depend on physical association of splicing factors with the polymerase.

Coupling of transcription and splicing is an intricate process, requiring coordinated action between the transcription complex and the spliceosome, a highly dynamic ribonucleoprotein megaparticle that catalyzes selective intron removal from newly synthesized transcripts.^14,15^ In each splicing cycle participate an estimated 150–200 proteins and five small nuclear RNAs (U1, U2, U4, U5 and U6 snRNAs)^15^; the later are incorporated into five structurally distinct ribonucleoprotein (snRNP) particles with distinct functions in spliceosome assembly and splicing catalysis. Exon/intron definition by U1 and U2 snRNPs stimulates binding of a pre-assembled U4/U6.U5 snRNP tri-particle. Following extensive conformational rearrangements and U1/U4 displacement, the two-step splicing reaction is catalyzed by the mature, catalytically active spliceosome composed of U2, U5 and U6 snRNPs. In addition to snRNPs, numerous accessory proteins participate in recognition of regulatory splicing sequences on the nascent transcript and in the continuous spliceosome remodeling.^15,16^

Complexity of the splicing reaction is further enhanced by the fact that the vast majority of pre-mRNAs can be alternatively spliced to produce multiple mRNA variants from a single gene, expanding thus protein diversity.^17^ Consequently, numerous mechanisms have evolved to ensure that the splicing machinery operates with a single nucleotide precision while maintaining the required plasticity for selective exon inclusion.^18^ These range from the presence of cis-acting elements on the transcript (splicing enhancers and silencers), to post-translational modifications of spliceosomal proteins, subject to regulation by intracellular and environmental cues.^16,19^ Splicing factor modifications can modulate their availability via changes in intracellular distribution, and modify interactions within the spliceosome but also with the transcription machinery.^20,21^ Reciprocally, posttranslational modifications of RNAPII (e.g. CTD phosphorylation) can influence splicing decisions through changes in the elongation rate of RNAPII (kinetic coupling) and via association of splicing regulators with RNAPII (recruitment coupling).^21^ It should be noted that the CTD of RNAPII appears to be primarily responsible for the communication of elongating complexes with the splicing machinery as it has been shown to regulate both constitutive and alternative RNA splicing.^21^

Functional coupling between transcription and RNA splicing can also be achieved indirectly, through the higher order chromatin structure.^22,23^ Chromatin compaction controls RNAPII transcription rates, while specific histone marks and chromatin remodelers can recruit splicing factors to nascent transcripts.^18,23^ An even more complex mechanism is triggered by template-DNA damage. Helix-distorting DNA lesions, physically arrest elongating RNAPII,^24^ cause wide-spread changes in chromatin structure,^24^ influence nuclear transport of splicing regulators,^4,20^ promote dissociation of spliceosomes from nascent transcripts and cause redistribution of splicing factors into nuclear speckles.^7^ These events result in a broad range of damage-induced alternative splicing (AS) events that can be attributed to changes in both the kinetic and the recruitment coupling between RNAPII and the splicing machinery.^4-6^ Additionally, as we recently reported, a substantial number of damage-induced AS events are under the control of ATM signaling.^7^ This latter observation further supports the notion that the functional coupling of transcription and splicing, receives cues from signaling systems that coordinate the cellular response.^18^

### DNA helix-distorting lesions and elongating RNA polymerase

Helix-distorting lesions on the template DNA can inhibit RNAPII progression and halt transcription until repaired by the specialized Transcription-Coupled Nucleotide Excision Repair (TC-NER) pathway.^24^ TC-NER initiates by stalling of RNAPII on a DNA lesion and depends on CSA, CSB and UVSSA for the assembly of a multiprotein repair complex and subsequent lesion removal and transcription resumption.^24,25^

Lesion-stalled RNAPII, with a footprint of ∼30 nucleotides around the lesion,^26^ creates a significant problem to the TC-NER system by limiting damage access. Structural studies of RNAPII trapped on helix-distorting lesions demonstrate that, despite mechanistic differences, all types of damage studied thus far (i.e. UV-induced cyclobutane pyrimidine dimers (CPDs)^27^ and various platinum adducts^28,29^) pose strong impediments to RNAPII forward translocation. An intrinsic property of RNAPII is its propensity to translocate in reverse (backtrack) when forward translocation is disfavored by DNA topology, or after nucleotide misincorporation.^30-32^ Thus, the prevailing model for damage access and TC-NER-complex assembly, postulates that RNAPII backtracks to expose the lesion.^33^ It should be noted that alternative pathways, such as degradation of RNAPII and translesion bypass can occasionally occur,^34^ but are considered to be last-resort mechanisms, functioning only under certain conditions for difficult to repair lesions.^25,33^

RNAPII operates in a dynamic environment where chromatin is relaxed in front of RNAPII, to facilitate transcription, and subsequently repressed behind the elongation complex. Compact nucleosomes pose strong impediments to forward translocation^35^ and, likely, also to a polymerase that translocates in reverse. Chromatin decompaction through acetylation, stimulates TC-NER and it is tempting to speculate that the observed recruitment of chromatin remodelers and modifiers (SNF2H/SMARCA5, p300 and HMGN1) facilitate chromatin relaxation and RNAPII backtracking.^24,33^ In addition, association of megadalton spliceosomes with pre-RNAs may also impede RNAPII backtracking, through steric hindrance. In response to transcription-blocking lesions, late stage spliceosomes (composed by U2, U5 and U6 snRNPs) dissociate from elongating RNAPII and are rapidly excluded from DNA damage sites.^7^ This dissociation is triggered by RNAPII pausing and may be a critical recruitment for its backtracking and subsequent lesion access by TC-NER.

### Co-transcriptional R-loops; from threats to genome instability to activators of DDR signaling

In the process of transcription, unwinding of the DNA helix creates positive and negative torsional strain ahead and behind the elongating RNAPII, respectively. Negative strain facilitates DNA-strand separation and back-hybridization of nascent RNA with complementary ssDNA adjacent to the transcription bubble; this results in a three nucleic acid structure (R-loop) containing a DNA:RNA hybrid across a region of unpaired DNA.^36^ R-loop formation is further favored by strand nicks and intrinsic DNA features, such as high GC content. Under physiological conditions R-loops are frequently formed throughout the genome, particularly in promoter proximal regions and 3′ UTRs of genes with high GC skew, where they function in transcription activation and termination, respectively.^37,38^ Interestingly, these regions are also enriched in paused elongating complexes, raising the possibility that prolonged pausing of RNAPII promotes R-loop formation, analogous to lesion-stalled RNAPII.^36^

Persistent R-loops are genotoxic^36,39,40^ as they can interfere with transcription and replication, increase the probability of replication fork collapse after collisions with stalled transcription complexes, and promote unscheduled replication by transcription associated recombination. Additionally, ssDNA in the R-loop is sensitive to mutagens, prone to formation of secondary structures such as G quadruplexes, can undergo spontaneous hydrolysis and Activation-Induced cytidine Deaminase (AID)-catalyzed modifications. To counteract R-loop toxicity, cells have evolved mechanisms to resolve and/or prevent unscheduled formation.^37^ Once formed, R-loops can be resolved by specialized RNA hydrolases (RNaseH1 and H2) or helicases (e.g., Pif1, DHX9, Senataxin) that unwind the RNA:DNA hybrid. Relaxation of superhelicity by DNA Topoisomerase I, RNA packaging into ribonuceloprotein particles and the coupling of transcription to RNA processing, prevent R-loop formation ^36,37,39,40^; the latter by a dual mechanism that depends on steric hindrance of hybridization and reduction of complementarity between intron-containing DNA and nascent (spliced) RNA.^36,37,39^ In support, R-loop accumulation and genomic instability have been described in yeast mutants deficient in RNA packaging and nuclear export proteins, and in metazoan cells after splicing factor depletion.^7,20,36,41^ Transcription-blocking DNA lesions can also promote R-loop formation through a mechanism that depends on RNAPII pausing and displacement of spliceosomes at sites of damage.^7^ Intriguingly, we found that R-loops formed after spliceosome displacement activate ATM-dependent DDR signaling and thus, disclosed a novel mechanism of ATM activation in response to transcription-blocking DNA damage.^7^

### ATM activation by transcription-blocking lesions; converting stochastic damage signals into an organized response

The ATM kinase is a master coordinator of the DDR, best described for its role in orchestrating an extensive signaling network in response to DNA double strand breaks (DSBs).^42^ Through this network ATM targets hundreds of proteins with specialized functions in damage repair, establishment of cell-cycle checkpoints and modulation of cellular metabolism and viability.^42,43^ Canonical ATM activation initiates after DSB detection by the MRE11–RAD50–NBS1 (MRN) complex, end requires MRN-mediated ATM anchoring to DNA-ends,^44^ TIP60/KAT5-mediated acetylation,^45^ ATM autophosphorylation, and monomerization^46^ of the previously inactive ATM dimer. The majority of active ATM molecules remain at DSBs where, together with their immediate targets, form structures known as DNA damage foci. Despite the robust activation of ATM by DSBs, the role of ATM in cellular homeostasis is not restricted to DSBs signaling. ATM coordinates the cellular response to structural chromatin changes and many types of stress (hypoxia, hyperthermia, oxidative stress), which activate ATM through MRN-independent pathways and in absence of detectable DSBs.^42,47^ It is noteworthy that under these conditions, active ATM does not localize in distinct foci (as after DSB induction) but is dispersed throughout the nucleus where it may access additional targets.

Transcription-blocking lesions stimulate R-loop formation and activate ATM via DSB-dependent and independent mechanisms. In replicating cells, R-loops have been associated with ATM activation and genomic instability through replication fork collapse,^36,48^ or when SSBs created during NER-dependent R-loop processing are converted to DSBs.^40^ We found that in non-replicating cells ATM can be activated by UV-photolesions via a non-canonical, DSB-independent but R-loop-dependent mechanism. Inhibition of spliceosome assembly or impaired resolution of R-loops, in absence of DNA damage, can also activate ATM indicating a role of ATM in signaling the presence of aberrant DNA: RNA structures.^7^

Active ATM has a plethora of targets. In addition to well described pathways such as p53, more than 1000 proteins, including numerous splicing factors, have been identified as potential ATM substrates.^49^ We found that in UV irradiated cells, ATM regulates core spliceosome remodeling and is responsible for 40% of UV-induced AS events genome-wide. Additionally, ATM controls a remarkable fraction of the UV-induced transcriptome, presumably through activation of its canonical target pathways. In this manner, stochastic DNA damage is “translated” into an organized ATM-controlled cellular response, establishing ATM as a major regulator of DDR to transcription-blocking lesions and the RNA splicing machinery as one of its key targets.

## A model of bidirectional coupling between splicing and ATM signaling, in response to transcription-blocking DNA lesions

**Figure 1.**
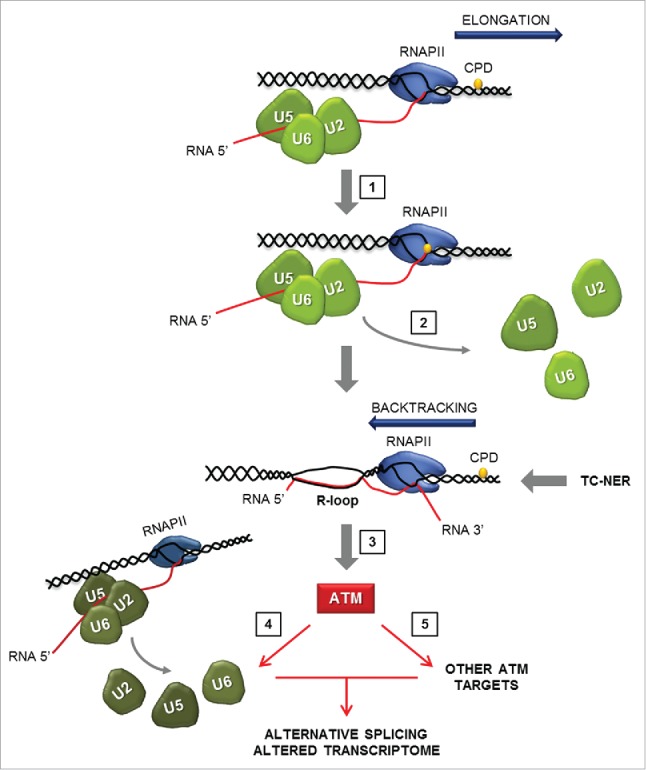
Model of reciprocal regulation between ATM and the core spliceosome When an elongating RNAPII encounters certain DNA lesions in the transcribed strand (such as UV-induced CPDs), forward translocation is blocked and the polymerase needs to backtrack to allow lesion access by the TC-NER system. RNAPII pausing triggers disassembly of U2/U5/U6 snRNP-containing spliceosomes from the pre-mRNA, as to alleviate steric hindrance and allow for backtracking of RNAPII and damage repair. Negative supercoiling behind RNAPII, in combination with the presence of spliceosome-free, intron-containing pre-mRNA, facilitates R-loop formation which results in non-canonical ATM activation. ATM signals to activate its target DDR pathways and mobilize spliceosomes from RNAPII located distal to the DNA lesion. ATM signaling culminates in wide-spread alternative splicing and gene expression changes. Key steps of the pathway are indicated and are as follows: **1**. RNAPII pausing when CPD enters its catalytic site; **2**. Spliceosome displacement; **3**. R-loop formation and ATM activation; **4**. ATM-dependent spliceosome displacement in-trans **5**. Activation of ATM-dependent DDR pathways. (U2, U5, U6: Small Nuclear Ribonucleoprotein Complexes; CPD; Cyclobutane Pyrimidine Dimers; RNAPII: RNA Polymerase II; TC-NER: Transcription-Coupled Nucleotide Excision Repair).

We have identified a novel, replication-independent, ATM activating mechanism and proposed a bidirectional coupling model (**Fig. 1**), in which spliceosome displacement from lesion-arrested RNAPII results in R-loop formation and subsequent ATM activation which signals in a feed-forward fashion to regulate spliceosome dynamics and AS genome-wide.^7^

Transcription-blocking DNA lesions induce rapid displacement from damaged areas (within seconds after irradiation) exclusively of U2, U5 and U6 snRNP factors. Spliceosomal snRNPs associate with pre-mRNAs at different stages of the splicing cycle and for different time periods; U1 and U4 have transient functions and we found that their levels of chromatin association (intrinsically lower) and mobility (inherently higher) are not significantly affected by irradiation.^7^ Under physiological conditions, late-stage spliceosomes containing U2, U5 and U6 snRNPs, form stable interactions and remain associated with the transcript for prolonged periods.^7^ Thus there is a higher probability that, when RNAPII encounters a transcription-blocking DNA lesion, a mature spliceosome is located at near proximity. The CPD in the transcribed strand slowly passes a translocation barrier and enters the polymerase active site where the CPD 5′-thymine preferentially directs uracil misincorporation, thereby blocking forward translocation. In the stalled complex, the lesion is inaccessible, and the polymerase conformation unchanged.^27^ Nucleotide misincorporation stimulates RNAPII backtracking, initially by one nucleotide, so that the elongation complex adopts an altered conformation state.^32^ It is likely that this altered structure disrupts interactions between elongation complexes and the splicing machinery and promotes its release, while at the same time prevents any further assembly. Formed spliceosomes are likely to pose steric hindrance to the removal/backtracking of RNAPII and their displacement may be required for damage access by the NER machinery and consequently, for efficient repair.

Displacement of co-transcriptional spliceosomes, in combination with negative supercoiling behind lesion-arrested RNAPII, facilitates hybridization of intron-retaining pre-mRNA with template DNA adjacent to the transcription bubble.^7^ In non-replicating cells the resulting R-loop activates ATM via DSB-independent mechanism(s) as evidenced by its dispersed localization throughout the nucleus which is comparable to treatments that activate ATM through changes in higher order chromatin.^7,42^ Intriguingly, R-loops are also known to influence chromatin structure,^36,50^ raising the possibility that the altered chromatin microenvironment in the vicinity of an R-loop, may be a sufficient trigger for ATM activation.

In support of the bidirectional coupling model, active ATM signals to further mobilize spliceosomes,^7^ presumably by destabilizing their interaction with nascent transcripts. Unlike spliceosome displacement from lesion-arrested RNAPII, which does not require ATM activity and is a stochastic event, the ATM-dependent spliceosome mobilization is an organized response, possibly to prohibit spliceosome occupancy on transcripts attached to polymerases located distal to DNA lesions and thereby, avoid additional transcriptional stress. A substantial body of experimental evidence supports the existence of splicing-dependent transcriptional checkpoints that reduce RNAPII elongation rates when spliceosome assembly is prevented.^22,51,52^ It is thus likely that, the ATM-dependent spliceosome mobilization could also result in RNAPII pausing as to prevent collision between the arrested and reinitiated transcription complexes. In addition, ATM signals through its other effectors to orchestrate the DDR, in part by imposing wide-spread gene expression changes. The importance of this dual role of ATM in the UV-induced transcriptome is highlighted by the large number of gene expression and alternative splicing changes that we identified to depend on ATM activity.^7^ Thus, ATM may coordinate the cellular response to transcription-blocking lesions by a regulatory strategy which acts at the genome-wide level to control splicing and transcription profiles, and at the gene level, possibly to prevent further cycles of transcription that could hamper DNA repair mechanisms.

The reciprocal regulation between ATM and the spliceosome mechanistically links these fundamental cellular processes, and establishes a novel, replication-independent role for ATM in regulating gene expression and alternative splicing in the presence of transcription-blocking DNA damage. It is tempting to speculate that this ATM function is particularly critical for post-mitotic tissues and may account, in part, for the progressive neurological phenotype of Ataxia Telangiectasia (AT) patients, in which mutant or absent ATM cannot trigger a proper cellular response to the gradual accumulation of metabolically derived (or endogenously produced) DNA lesions.^42,43^ Interestingly, AT patients share clinical features with TC-NER-deficiency syndromes, thought to derive from accumulation of unresolved lesion-arrested transcription complexes.^24^ In this respect, it is intriguing that neurological function is also impaired in patients with disrupted R-loop metabolism (e.g. ataxia oculomotor apraxia type 2, amyotrophic lateral sclerosis type 4, trinucleotide repeat expansion disorders)^37,38,53^ and patients with splicing deficiencies (e.g., spinal muscular atrophy).^19,54^ In response to DNA damage, these pathways are functionally linked ex-vivo^7^; whether they are also linked in vivo would be a challenging question to address.
